# 20-hydroxyecdysone (20E) signaling regulates amnioserosa morphogenesis during *Drosophila* dorsal closure: EcR modulates gene expression in a complex with the AP-1 subunit, Jun

**DOI:** 10.1242/bio.058605

**Published:** 2021-08-17

**Authors:** Byoungjoo Yoo, Hae-yoon Kim, Xi Chen, Weiping Shen, Ji Sun Jang, Shaianne N. Stein, Olga Cormier, Lionel Pereira, Claire R. Y. Shih, Charles Krieger, Bruce Reed, Nicholas Harden, Simon J. H. Wang

**Affiliations:** 1 Department of Molecular Biology and Biochemistry, Simon Fraser University, 8888 University Drive, Burnaby, BC V5A 1S6, Canada; 2 Department of Biology, University of Waterloo, 200 University Avenue West, Waterloo, ON N2L 3G1, Canada; 3 Department of Biomedical Physiology and Kinesiology, Simon Fraser University, 8888 University Drive, Burnaby, BC V5A 1S6, Canada

**Keywords:** 20-hydroxyecdysone (20E), Ecdysone receptor (EcR), Jun, Zipper (Zip), Amnioserosa, Dorsal closure

## Abstract

Steroid hormones influence diverse biological processes throughout the animal life cycle, including metabolism, stress resistance, reproduction, and lifespan. In insects, the steroid hormone, 20-hydroxyecdysone (20E), is the central hormone regulator of molting and metamorphosis, and plays roles in tissue morphogenesis. For example, amnioserosa contraction, which is a major driving force in *Drosophila* dorsal closure (DC), is defective in embryos mutant for 20E biosynthesis. Here, we show that 20E signaling modulates the transcription of several DC participants in the amnioserosa and other dorsal tissues during late embryonic development, including *zipper*, which encodes for non*-*muscle myosin. Canonical ecdysone signaling typically involves the binding of Ecdysone receptor (EcR) and Ultraspiracle heterodimers to ecdysone-response elements (EcREs) within the promoters of responsive genes to drive expression. During DC, however, we provide evidence that 20E signaling instead acts in parallel to the JNK cascade via a direct interaction between EcR and the AP-1 transcription factor subunit, Jun, which together binds to genomic regions containing AP-1 binding sites but no EcREs to control gene expression. Our work demonstrates a novel mode of action for 20E signaling in *Drosophila* that likely functions beyond DC, and may provide further insights into mammalian steroid hormone receptor interactions with AP-1.

## Abstract (precis)

**Summary:** During *Drosophila* dorsal closure, 20E signaling acts non-canonically through an interaction between EcR and the AP-1 subunit, Jun, to control gene expression at regions containing AP-1 motifs but no EcREs.

## INTRODUCTION

Dorsal closure (DC) of the *Drosophila* embryo is a developmental wound-healing event in which a hole in the dorsal epidermis, occupied by a transient epithelium, the amnioserosa, is closed by migration of the epidermal flanks (reviewed in Harden, 2002). DC serves as a paradigm for morphogenetic events where tissues are brought together and fused, including the vertebrate processes of embryonic neural tube closure and palate fusion. A recurring finding in studies of wound healing and developmental epithelial closures is that cells occupying the hole contribute to closure by contracting in response to signaling from the hole margin by transforming growth factor β (TGF-β) superfamily ligands (reviewed in Belacortu and Paricio, 2011). This mechanism is conserved in DC where the leading edge epidermal cells (i.e. the dorsal-most epidermal, DME, cells) secrete Decapentaplegic (Dpp), a TGF-β ligand that activates a signaling pathway in the amnioserosa through the receptors Thickveins (Tkv) and Punt, which are required for correct amnioserosa morphogenesis (Fernández et al., 2007; Wada et al., 2007; Zahedi et al., 2008). Recent studies suggest that autonomous contraction of the amnioserosa alone can drive DC and it is of interest to know how this is initiated (Pasakarnis et al., 2016; Wells et al., 2014). One way that synchronized contraction of the amnioserosa cells could be achieved is through an autocrine signaling process in which the amnioserosa cells produce a secretable ligand that induces their own contraction. In a search for such a pathway downstream of Dpp in the amnioserosa, we considered signaling by the steroid hormone, 20-hydroxyecdysone (20E). The amnioserosa is a major source of 20E during embryogenesis, and mutants of the Halloween group of genes, which encode enzymes in the 20E biosynthetic pathway, display DC defects (Chavez et al., 2000; Giesen et al., 2003; Kozlova and Thummel, 2003; Niwa et al., 2010; Ono et al., 2006).

Canonical ecdysone signaling involves the binding of 20E-activated Ecdysone receptor (EcR) and Ultraspiracle (Usp) heterodimers to ecdysone-response elements (EcREs) to promote gene expression (Dobens et al., 1991; Yao et al., 1993). Here, we show that 20E modulates gene expression in the amnioserosa and other dorsal tissues in a novel manner. Key DC participants in the DME cells and amnioserosa are transcribed in response to a c-Jun N-terminal kinase (JNK) MAPK cascade operating through the AP-1 transcription factor, which consists either as a homodimer of Jun or a heterodimer of Jun and Fos (Ríos-Barrera and Riesgo-Escovar, 2013). We present evidence that 20E signaling acts in parallel to the JNK cascade by regulating Jun through the activation of EcR, which carries Jun from the cytoplasm to genomic regions containing AP-1 binding sites but no EcREs in DC genes. To our knowledge, this the first time that EcR has been shown to directly interact with AP-1 in *Drosophila*, though a genetic interaction has been recently uncovered during the pruning of sensory neuron dendrites (Zhu et al., 2019). Our work demonstrates a mechanism for fine tuning the output from the JNK cascade during DC, and reveals an alternative mode of action for 20E signaling that likely functions beyond DC, as several mammalian steroid hormone receptors can also regulate gene expression in a complex with AP-1 (reviewed in Marino et al., 2006).

## RESULTS

### Dpp signaling to the amnioserosa leads to 20E production, which is required for correct morphogenesis of the tissue during DC

**Fig. 1.**
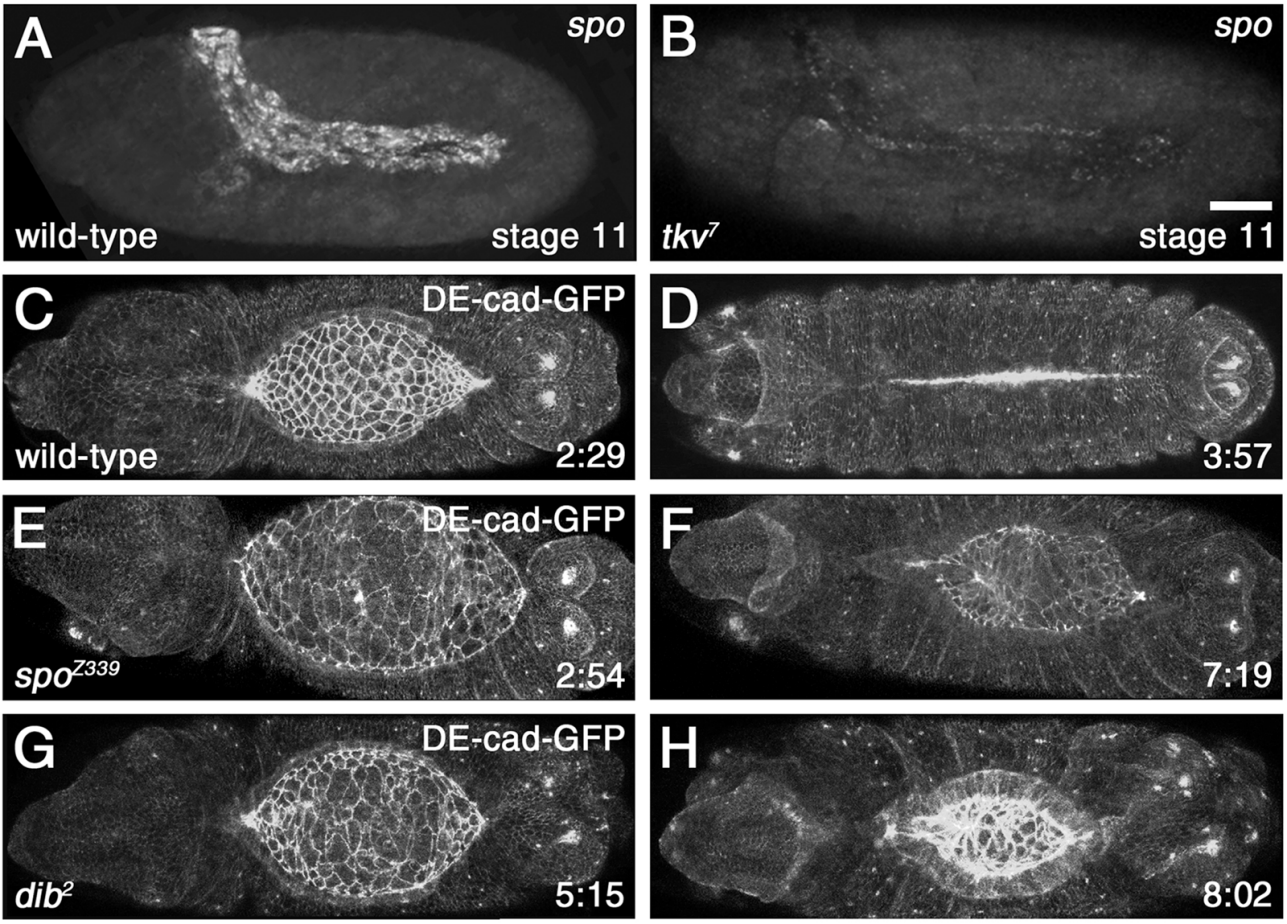
**Dpp signaling is required for the expression of *spo*, which, together with another gene involved in 20E biosynthesis, *dib*, is required for correct morphogenesis of the amnioserosa during DC.** (A) FISH showing *spo* expression in the amnioserosa during germband retraction in wild type. (B) In embryos mutant for the Dpp receptor, Tkv, *spo* expression is lost. (C-H) Stills from live imaging of DC-staged wild type (C,D), *spo* mutant (E,F), and *dib* mutant (G,H) embryos, showing uniform amnioserosa morphogenesis and closure of the epidermis in wild type (see Movie 1), but defective amnioserosa morphogenesis and failure of DC in *spo* and *dib* mutant embryos (see Movies 2 and 3). A *ubi-DE-cadherin-GFP* transgene was expressed in all embryos to visualize morphology. Time points (h:min) are shown in the bottom right corner of each panel. Scale bar: 50 µm (B).

Given that Dpp signaling to the amnioserosa is required for morphogenesis during DC, and that 20E required for DC is produced in the amnioserosa, we tested the hypothesis that Dpp regulated 20E production. An attractive mechanism for the timely production of 20E in the amnioserosa could be through the presence of all but one or two of the biosynthetic pathway members in the amnioserosa. According to this model, 20E production could be activated specifically in the amnioserosa through tissue-specific transcriptional regulation of just a couple of the pathway members. The *spook* (*spo*) gene is the only locus encoding a member of the 20E biosynthetic pathway known to be transcribed in the amnioserosa, although other members of the pathway are expressed in the amnioserosa anlage (Ono et al., 2006). In *tkv^7^* mutant embryos, *spo* expression detected by fluorescent *in situ* hybridization (FISH) was largely abolished (Fig. 1A,B). If 20E is required for morphogenesis during DC, then mutants in 20E production should show morphogenetic defects. Indeed, live imaging of embryos mutant for *spo* or *disembodied* (*dib*), another enzyme in the 20E biosynthetic pathway, revealed abnormalities in amnioserosa morphogenesis and a failure to complete DC properly (Fig. 1C-H and Movies 1-3). In particular, mutants lacking 20E showed uneven contractility of the amnioserosa cells and a failure to complete amnioserosa morphogenesis, suggesting perturbation of cytoskeletal regulation. Thus, candidate genes for regulation by 20E during DC are likely regulators or components of the cytoskeleton expressed in the amnioserosa.

### The timing of expression of four JNK-responsive genes in the amnioserosa is regulated by 20E signaling

Three JNK-responsive genes were previously found to be expressed at high levels in the DME cells and amnioserosa during DC: *jaguar* (*jar*), *jupiter* (*jup*), and *Z band alternatively spliced PDZ-motif protein 52* (*zasp52*) (Ducuing et al., 2015). The duration of expression varies from gene to gene, and we determined by FISH that this was due to transcriptional regulation (Fig. S1A-L). *jar* and *zasp52* have been shown to be required for scar-free DC (Ducuing and Vincent, 2016; Millo et al., 2004), while *jup* encodes for a little-studied microtubule-associated protein (Karpova et al., 2006). *zipper* (*zip*) encodes for non-muscle myosin, which is required for cell shape change during DC, and is transcribed in a similar pattern to these three genes (Fig. S1M-P) (Franke et al., 2005; Young et al., 1993; Zahedi et al., 2008). To test if *zip* was also a JNK-responsive gene, *prd-GAL4* was used to drive segmental embryonic expression of either an activated version of the small Rac1 GTPase, which activates the JNK pathway (Glise and Noselli, 1997; Hou et al., 1997), or a constitutively active form of JNKK encoded by *hemipterous* (*hep*) (Weber et al., 2000). Ectopic expression of Rac1V12 or Hep^CA^ both resulted in elevated *zip* transcripts in *prd* stripes in the epidermis and amnioserosa (Fig. S1Q-S), indicating regulation of *zip* expression by JNK signaling. We confirmed that endogenous JNK signaling was required for this process by impairing the pathway through expression of Bsk^DN^, a dominant negative form of JNK encoded by *basket* (*bsk*) (Weber et al., 2000), which resulted in a loss of *zip* transcripts in *prd* stripes in the DME cells (Fig. S1T).

**Fig. 2.**
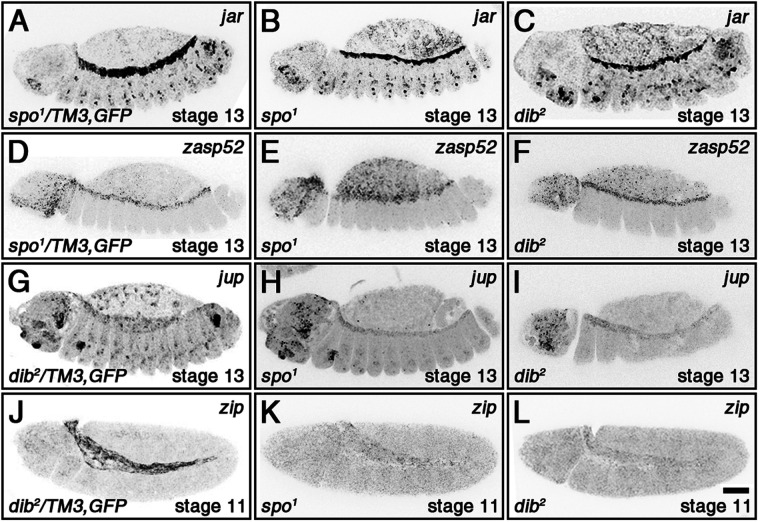
***spo* and *dib* regulate the expression of JNK-responsive genes in amnioserosa and DME cells.** For clearer views of the changes in gene expression, representative images have been inverted. Heterozygous siblings of the homozygous mutant embryos served as controls for each FISH stain, as they were treated under identical conditions within the same tube. (A-C) *jar* expression in the amnioserosa shuts off by the start of DC in the control (A), but persists in a subset of amnioserosa cells in both *spo* and *dib* homozygous mutants (B,C) (see Fig. S2A,B for quantifications). Effects in the DME cells were not readily observable (data not shown). (D-F) Similar results were observed for *zasp52* (see Fig. S2C,D for quantifications). (G-I) Expression of *jup* persists in the amnioserosa during DC in the control (G), but is significantly reduced in both *spo* and *dib* homozygous mutants (H,I) (see Fig. S2E,G for quantifications). A slight but statistically significant decrease in expression within the DME cells was also observed in the homozygous mutants (see Fig. S2F,H for quantifications). (J-L) *zip* is strongly expressed in the amnioserosa during germband retraction (J), but is lost in both *spo* and *dib* homozygous mutants (see Fig. S2I,K for quantifications). A slight but statistically significant decrease in expression within the DME cells was also observed in the homozygous mutants (data not shown; see Fig. S2J,L for quantifications). Scale bar: 50 µm (L). DME cells=dorsal-most epidermal cells, which flank the amnioserosa.

We next used FISH to examine the expression patterns of the four JNK-responsive genes in embryos mutant for either *spo* or *dib* to determine if loss of 20E also had an effect on their transcription. *jar* and *zasp52* expression normally disappeared from the amnioserosa by the beginning of DC in *spo^1^* and *dib^2^* heterozygous mutant embryos (Fig. 2A,D), which served as controls that displayed similar expression patterns to wild type (Fig. S1C,K). However, expression of both genes persisted in a subset of amnioserosa cells in *spo^1^* and *dib^2^* homozygous mutant embryos undergoing DC (Fig. 2B,C,E,F). Effects in the DME cells were not readily observable. In contrast to *jar* and *zasp52*, *jup* and *zip* expression in the amnioserosa was shut off earlier in *spo^1^* and *dib^2^* homozygous mutants (Fig. 2H,I,K,L) than in controls (Fig. 2G,J; Fig. S1G,M for wild type). A small but statistically significant decrease in *jup* and *zip* expression within the DME cells was also observed in the mutants. Quantification of FISH signal can be found in the supplementary material (Fig. S2). Based on these results, we conclude that 20E signaling mainly regulates the timing of the expression of at least four JNK-responsive genes in the amnioserosa during DC.

### EcR forms a complex with the AP-1 transcription factor subunit, Jun, in amnioserosa nuclei

**Fig. 3.**
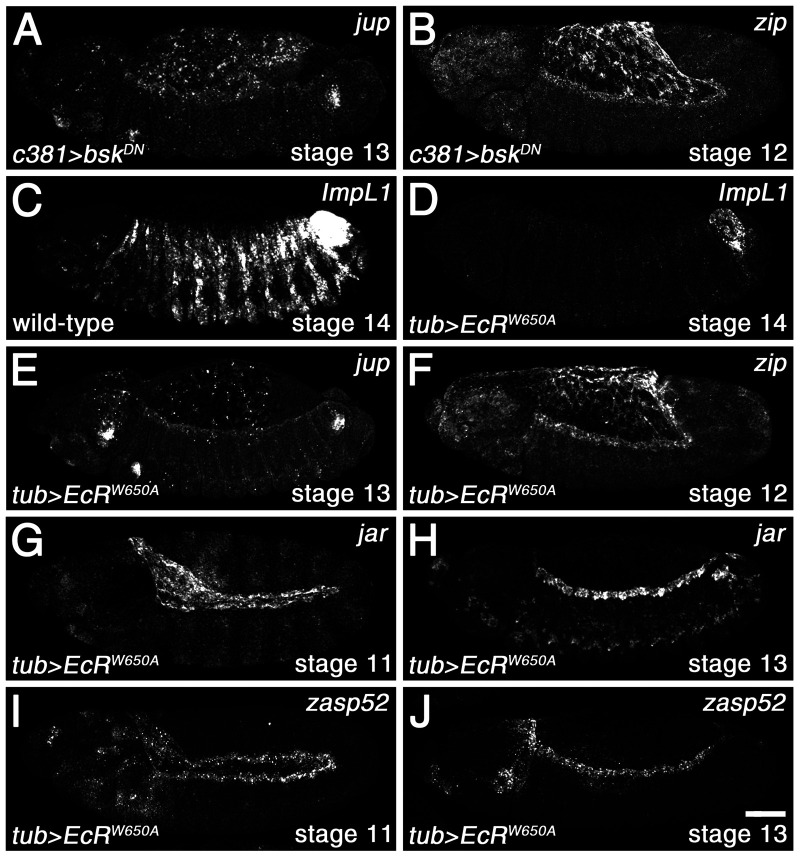
**20E-mediated gene expression in the amnioserosa is independent of the JNK and canonical ecdysone pathways.** (A,B) Impairment of the JNK pathway in the amnioserosa via Bsk^DN^ expression does not inhibit the transcription of *jup* during DC (A) or *zip* during mid-germband retraction (B). (C-J) Impairment of canonical ecdysone signaling through the ubiquitous expression of EcR-W650A, which prevents endogenous EcR from dimerizing with Usp, blocks transcription of the known ecdysone-responsive gene, *IMP-L1*, in the epidermis (C,D). However, similar to Bsk^DN^, EcR-W650A does not suppress *jup* (E) or *zip* (F) transcription in the amnioserosa. Furthermore, transcription in the DME cells remains unaffected. Transcription of *jar* and *zasp52* is also unaltered in the amnioserosa during early germband retraction (G,I) and in the DME cells during DC (H,J). Scale bar: 50 µm (J).

20E canonically activates EcR, which in turn forms a heterodimer with the nuclear receptor, Usp, and binds to EcREs in target genes to control expression (Dobens et al., 1991; Yao et al., 1993). EcR is structurally similar to the vertebrate estrogen receptor, which has been shown to be able to bind to AP-1, the transcription factor acting in the JNK cascade that is commonly composed of heterodimers of Jun and Fos (Marino et al., 2006). Interestingly, JNK signaling is shut off in the amnioserosa prior to DC, with Fos adopting a largely cytoplasmic distribution but with Jun retaining some nuclear localization (Reed et al., 2001). This downregulation of JNK signaling in the amnioserosa is required for DC, and we wondered whether there might be a ‘handing over of control’ of gene expression in the amnioserosa from the JNK pathway to 20E signaling through an interaction between Jun and EcR. In wild type, *jar* and *zasp52* lose amnioserosa expression by mid-germband retraction (Fig. S1B,J), whereas expression of *jup* and *zip* persist longer in the tissue (Fig. S1F,N). We expressed Bsk^DN^ in the amnioserosa to test for a requirement for JNK signaling in maintaining *jup* and *zip* transcription and found that it was not required (Fig. 3A,B). Ubiquitous expression of a dominant negative version of EcR, EcR-W650A, which is thought to block endogenous EcR from dimerizing with Usp and thereby repress expression at EcREs, failed to inhibit *jup* and *zip* transcription in both the amnioserosa and DME cells (Fig. 3E,F), but did block epidermal transcription of a known 20E-responsive gene, *ecdysone-inducible gene L1* (*IMP-L1*) (Fig. 3C,D) (Cherbas et al., 2003; Natzle et al., 1988, 1992). Additionally, no effects on *jar* and *zasp52* transcription were observed (Fig. 3G-J). These results indicate that expression of the four genes in the amnioserosa is not dependent on JNK or canonical ecdysone signaling.

**Fig. 4.**
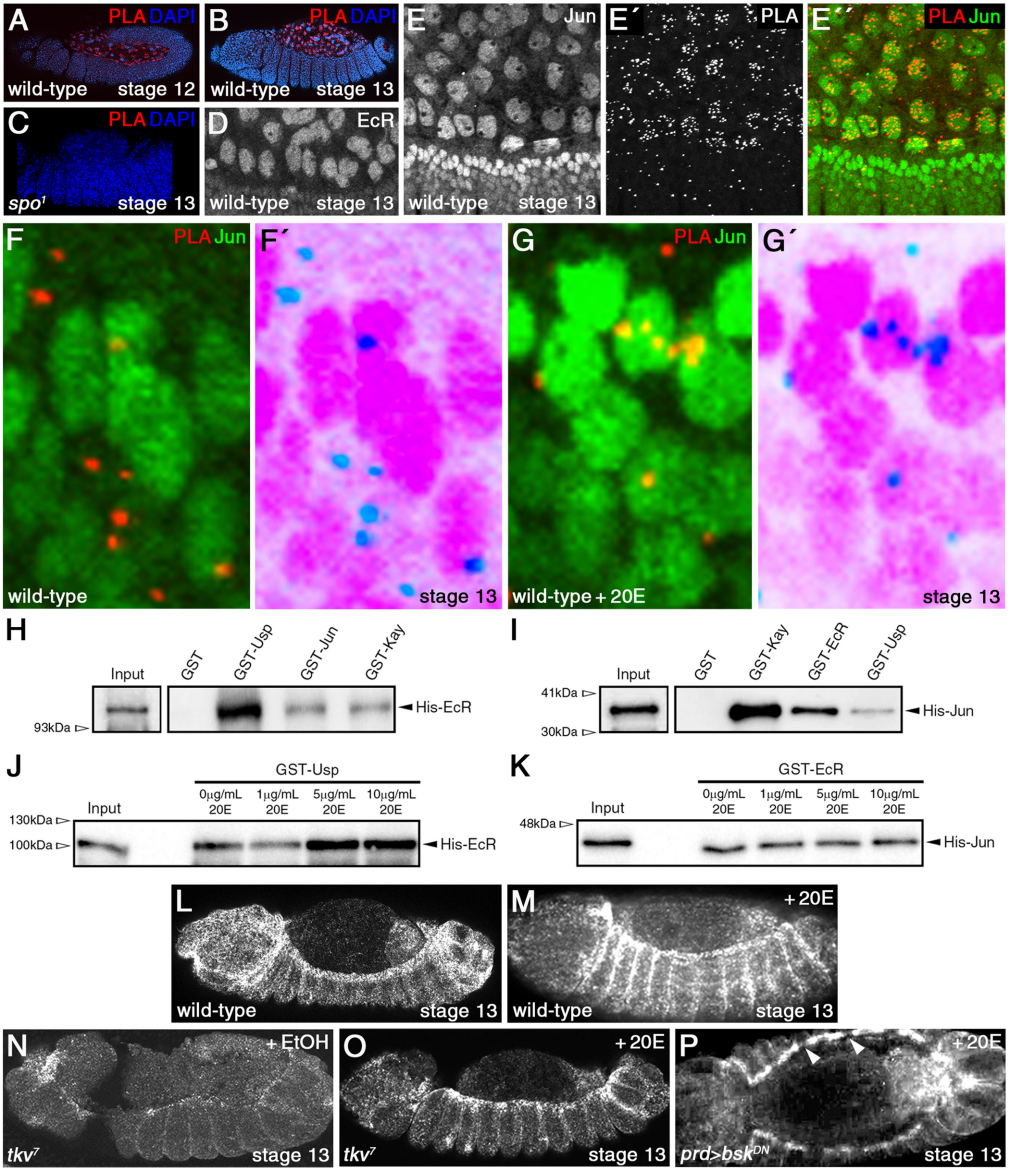
**Evidence of interactions between 20E signaling and the JNK pathway.** (A,B) Wild type embryos subjected to PLA between EcR/Jun (red) and stained with DAPI (blue) predominately show clusters of PLA complexes in amnioserosa nuclei during germband retraction (A) and DC (B). (C) PLA signals are not observed in *spo* mutant embryos. (D) Close-up view of a wild type embryo stained with anti-EcR antibody shows highest levels of EcR in amnioserosa nuclei during DC (this antibody was used in the PLA experiments). (E-E″) Close-up view of a wild type embryo subjected to PLA between EcR/Jun (E′, red in E″) and stained with anti-Jun antibody (E, green in E″). Highest levels of Jun are found in the DME cells, but Jun is also present in amnioserosa nuclei during DC. (F-G′) Similarly stained embryos as in E-E″. High magnification views of epidermal cells show that PLA complexes are largely cytoplasmic in wild type embryos (F,F′), where endogenous 20E levels are low, but translocate into the nucleus upon 20E-treatment (G,G′). F′ and G′ are inverted images. (H-K) Immunoblot analysis of pull-down assays between EcR and Jun. EcR immunoblots show that GST-Jun and GST-Kay (Fos) are both able to pull-down His-EcR (H). No binding was observed in the negative control, which involved GST alone. GST-Usp served as a positive control since Usp is known to dimerize with EcR. Jun immunoblots show that GST-EcR and GST-Usp were both able to pull-down His-Jun in reciprocal assays (I). No binding was observed with GST alone. GST-Kay (Fos), the other subunit of the AP-1 transcription factor, served as a positive control. Addition of 20E increases binding between EcR and Usp (J), but not EcR and Jun (K). All inputs represent 1%. His-EcR (expected size=97.4 kDa), His-Jun (34.9 kDa). (L,M) DC-staged wild type embryos treated with 20E show ectopic *zip* transcription in the epidermis (M) in comparison to untreated embryos (L). (N,O) DC-staged *tkv* mutant embryos show reduced *zip* transcript levels (N), but upon 20E-treatment, *zip* transcription is restored in the DME cells (O). (P) In contrast, 20E-treatment does not restore *zip* transcription in DME cells expressing Bsk^DN^ (arrowheads).

We wondered if 20E regulates gene expression in the amnioserosa by modulating an interaction between EcR and the AP-1 transcription factor subunit, Jun, given Jun's persistent nuclear localization in the tissue. We looked for such an *in vivo* interaction using proximity ligation assay (PLA) (Söderberg et al., 2006), and found that Ecr formed a complex with Jun predominately in the amnioserosa from germband retraction to DC (Fig. 4A,B). PLA signal was largely absent in *spo^1^* mutant embryos, indicating a loss of EcR-Jun complexes (Fig. 4C). For these experiments, we used antibodies against EcR and Jun that revealed their presence in amnioserosa nuclei, as well as in epidermal nuclei though EcR was much less abundant in comparison to Jun (Fig. 4D,E). Higher magnification views showed that complexes of EcR and Jun were largely found in amnioserosa nuclei, with much lower levels found throughout the epidermis (Fig. 4E′-G′). Negative controls that were performed with anti-EcR antibody omitted or with anti-Jun replaced by anti-phosphorylated Mothers against dpp (pMad), which detects another transcription factor participating in DC (reviewed in Affolter et al., 2001), showed very low signal background (Fig. S3). It is not surprising that multiple PLA signals are seen in the amnioserosa, as the amnioserosa is the site of high levels of 20E and nuclear EcR. We next assessed if the association between Ecr and Jun involved direct physical interaction using reciprocal GST pull-down assays and found that EcR could bind directly to Jun, *in vitro* (Fig. 4H,I; see Fig. S4 for relative levels of bait proteins used in the assays). These assays also showed that EcR could bind to Kayak (Kay, Fos in mammals) and Jun could bind to Usp, though further work is required to confirm the *in vivo* relevance of these interactions. Interestingly, addition of 20E did not increase binding between Jun and EcR (Fig. 4J,K).

In the embryonic epidermis, where 20E levels are lower, complexes of EcR and Jun were also observed but were consistently outside the nucleus, with 72.5% of 131 PLA signals counted in a wild type embryo being cytoplasmic (Fig. 4F,F′). Soaking embryos in 20E caused EcR-Jun complexes in the epidermis to translocate into the nucleus with only about a third of PLA signals remaining in the cytoplasm (Fig. 4G,G′), and this was accompanied by elevated expression of *zip* transcripts in the epidermis (Fig. 4L,M). Collectively, these results suggest that high levels of 20E promote the movement of EcR-Jun complexes into the nucleus where they can modulate gene expression.

### 20E signaling requires the JNK pathway to drive ectopic *zip* expression in the epidermis

Having determined that exogenous 20E can elevate *zip* expression in the embryonic epidermis, we explored the requirements for such regulation. We first assessed the ability of exogenous 20E to restore *zip* transcription in the DME cells of *tkv^7^* mutant embryos, in which endogenous 20E is absent, and found that it could (Fig. 4N,O). As seen above, knockdown of the JNK pathway in the amnioserosa through expression of Bsk^DN^ did not prevent 20E-dependent gene expression in that tissue. In contrast, exogenous 20E was incapable of restoring *zip* transcription in DME cells with Bsk^DN^ expression (Fig. 4P), indicating a requirement for JNK pathway activation in triggering 20E-induced ectopic expression of *zip* in the epidermis.

### Discovery of putative EcR-AP-1 binding regions in or near DC genes

**Fig. 5.**
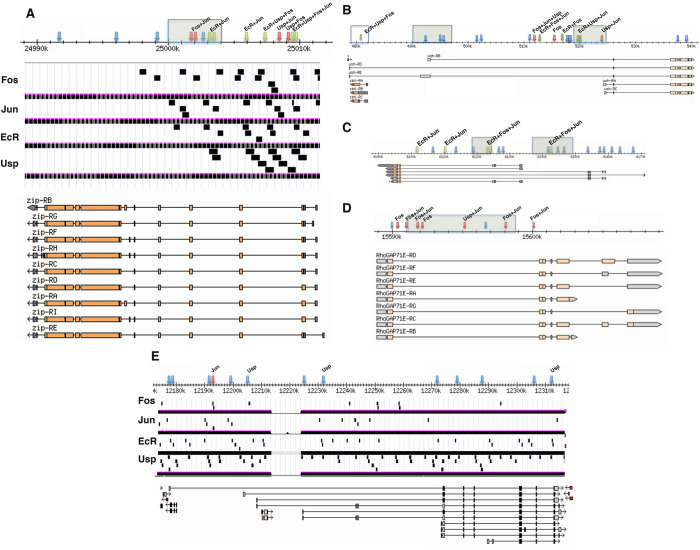
**Putative EcR-AP-1 binding regions are located in large introns of genes expressed in dorsal tissues during germband retraction and DC.** Diagrams are modified from GBrowse and UCSC Genome Browser (Gonzalez et al., 2021; Larkin et al., 2021). Arrows mark consensus AP-1 binding sites (TGANTCA). Blue arrows are sites that do not overlap with ChIP-seq peaks for EcR, Jun or Kay (Fos); red arrows are sites that overlap with ChIP-seq peaks for Jun and/or Kay (Fos); green arrows are sites that overlap with ChIP-seq peaks for EcR (ENCODE Project Consortium, 2012). Labels above arrows indicate additional ChIP-seq peaks, whereas shaded boxes are EcR binding regions identified by Gauhar and colleagues (ENCODE Project Consortium, 2012; Gauhar et al., 2009). Sample distributions of ChIP-seq peaks are denoted in panels A and E as black rectangles. (A) *zip* locus showing no binding of the four transcription factor proteins to exons. (B) *cbt* and *ush* genomic region. The unshaded box on the far left denotes sequences controlling *cbt* expression. (C) *EcR* genomic region. (D) *RhoGAP71E* genomic region. (E) Control large intron gene, *bruno 1* (*bru1*), showing distribution of consensus AP-1 binding motifs in a gene not known to be regulated by JNK or 20E signaling. There is only about one AP-1 binding motif every 10 kb.

We have shown that 20E is required for the expression of *zip* in the amnioserosa - is there any evidence of EcR directly binding to the *zip* locus? Gauhar and colleagues mapped 502 genomic binding regions for EcR-Usp in *Drosophila* Kc167 cells treated with 20E, one of which resides in intronic sequences of *zip* (Fig. 5A) (Gauhar et al., 2009). This region lacks a consensus EcRE but does contain five copies of the AP-1 binding motif consensus, TGANTCA, suggesting that EcR binds to the *zip* locus through its association with Jun. We wondered if this region constituted an enhancer modulating gene expression in the amnioserosa by EcR and Jun, and screened through the Kc167 EcR-Usp binding regions for those containing at least four consensus AP-1 binding motifs but no EcRE consensus site. We identified 51 additional genomic regions fitting these criteria (listed in Table S1). 22 of these regions are in or near genes that have previously been shown to be expressed in the amnioserosa. Interestingly, *EcR* was picked up in the screen, thus indicating a feedback loop. In an effort to look for further evidence of joint regulation of EcR and Jun in such genes, we used chromatin immunoprecipitation sequencing (ChIP-seq) data generated by Kevin White's lab as part of the ENCODE Project Consortium (Davis et al., 2018; ENCODE Project Consortium, 2012). These data include genome-wide binding regions for GFP-tagged versions of EcR, Usp, Jun, and Kay (Fos) immunoprecipitated with anti-GFP antibodies from white prepupae, 0-12 h old embryos, wandering third instar larvae, and 0-24 h old embryos, respectively. Putative binding regions for these proteins were scattered throughout *zip* introns, but not in open reading frames (Fig. 5A). Though regions associated with *jup*, *jar*, and *zasp52* were not picked up in the screen performed by Gauhar and colleagues, potential EcR binding sites were discovered by the ENCODE Project Consortium for *jup* and *jar*, but not *zasp52* (not shown) (ENCODE Project Consortium, 2012; Gauhar et al., 2009). Interestingly, Alexander Stark's group used self-transcribing active regulatory region sequencing (STARR-seq) to identify hormone-responsive transcriptional enhancers in S2 and ovarian somatic cells, and found a 20E-repressed enhancer within an intron of *zasp52* but no corresponding EcR binding site (Arnold et al., 2013; Shlyueva et al., 2014). Their study revealed that only 5.5% of the identified repressed enhancers had significant EcR ChIP-seq enrichment, indicating that 20E-mediated repression may involve a mechanism that is predominately independent of EcR binding.

To explore further the regulation of gene expression by EcR acting at AP-1 binding motifs, we selected five genes from our screen to determine if 20E also regulates their expression through FISH. The five genes were the known DC participants *cbt* and *ush*, plus *EcR*, *RhoGAP71E*, and *Mes2*. All of these genes are expressed in the amnioserosa (Fig. S5), and enriched in other dorsal tissues including the yolk sac and hindgut (*cbt*, Fig. S5A-C), the dorsal epidermis (*ush*, Fig. S5D-F), and the dorsal vessel (*RhoGAP71E*, Fig. S5J-L; *Mes2*, Fig. S5M-O) (Belacortu et al., 2011; Kozlova and Thummel, 2000; Lada et al., 2012).

**Fig. 6.**
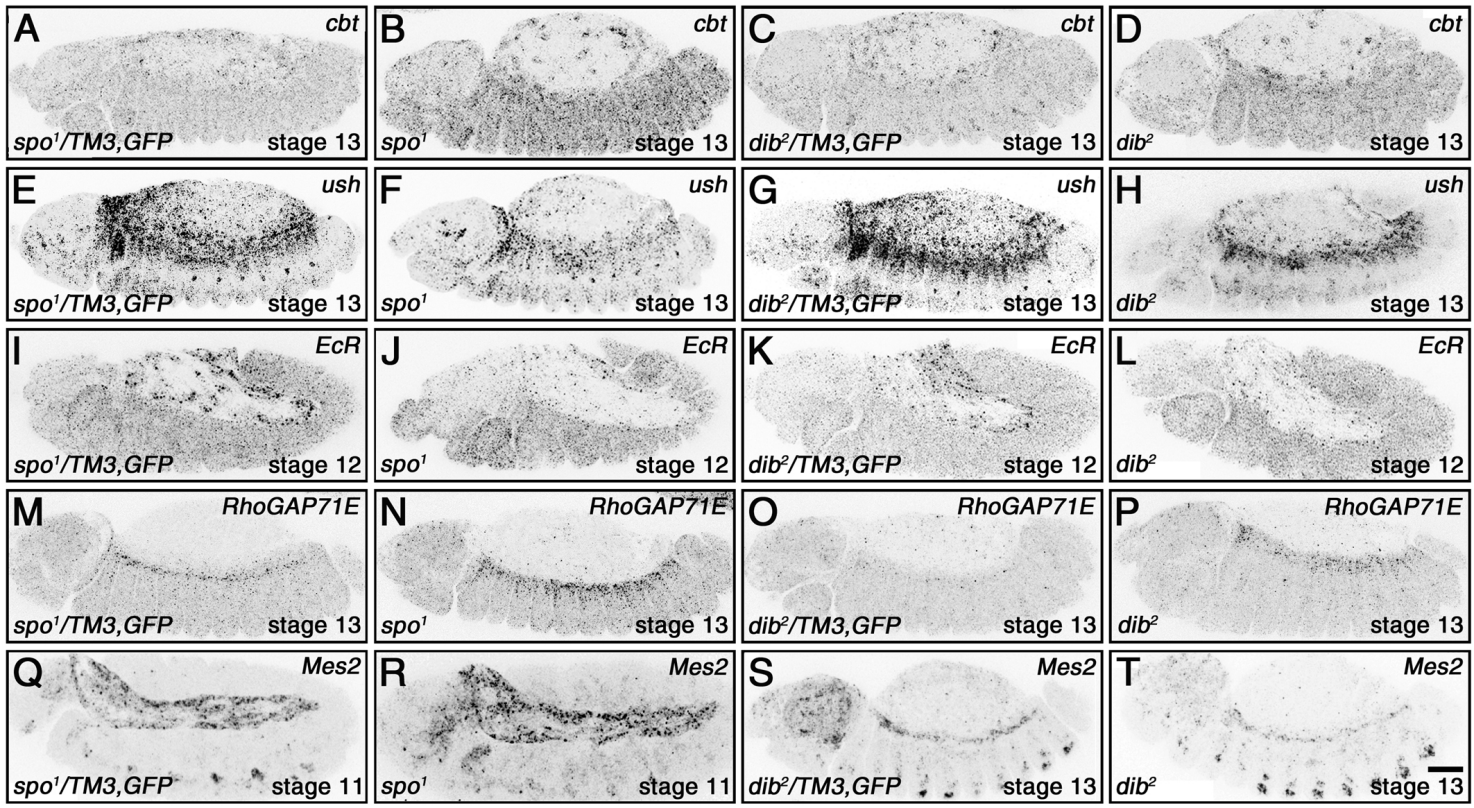
***spo* and *dib* regulate the expression of genes bearing putative EcR-AP-1 binding regions in dorsal tissues during germband retraction and DC.** For clearer views of the changes in gene expression, representative images have been inverted. Heterozygous siblings of the homozygous mutant embryos served as controls for each FISH stain, as they were treated under identical conditions within the same tube. (A-D) Relative to the controls (A,C), *spo* and *dib* mutant embryos both show increased *cbt* expression in the epidermis but no change in the amnioserosa (B,D) (see Fig. S5A-D for quantifications). (E-H) Control embryos have high levels of *ush* expression in both the peripheral amnioserosa cells and dorsal epidermis (E,G), but expression is reduced in both tissues of embryos mutant for either *spo* or *dib* (F,H) (see Fig. S5E-H for quantifications). (I-L) In contrast to the controls (I,K), expression of *EcR* in the amnioserosa during germband retraction is lost with disruption of 20E signaling (J,L) (see Fig. S5I,J for quantifications). (M-P) *RhoGAP71E* expression is restricted to the dorsal vessel in control embryos during DC (M,O), but is ectopically expressed in the dorsal epidermis in both *spo* and *dib* mutant embryos (N,P) (see Fig. S5 K,L for quantifications). (Q-T) No change in the expression of *Mes2* is observed between control (Q,S) and mutant (R,T) embryos (see Fig. S5M-P for quantifications). Scale bar: 50 µm (T).

*cbt* is located in an intron of *ush*, but transcribed in the opposite direction. Based on prior immunostains, Cbt is expressed in yolk sac nuclei, the amnioserosa, as well as in other more ventral tissues during DC (Belacortu et al., 2011). Expression in the yolk sac and amnioserosa appeared unperturbed in *spo^1^* and *dib^2^* mutant embryos, but relative to these two tissues, *cbt* transcript levels were elevated in the epidermis during DC (Fig. 6A-D; quantifications in Fig. S6A-D), indicating inhibition of epidermal *cbt* expression by 20E signaling. A previous study used reporter genes to identify a block of sequences that promoted expression in many of the tissues Cbt is found and likely constitutes the major control region for *cbt* (Belacortu et al., 2011). This region has a single AP-1 binding motif, which EcR and Fos have been shown to bind in the vicinity of (Fig. 5B) (ENCODE Project Consortium, 2012). Starting about 8 kb upstream of the *cbt* regulatory region is a stretch of about 35 kb of intronic sequences with multiple AP-1 binding motifs that putatively recruit various combinations of EcR, Usp, Jun, and Kay (Fos), and are likely control sequences for *ush* (Fig. 5B) (ENCODE Project Consortium, 2012). We found that the expression of *ush* in the peripheral amnioserosa cells and dorsal epidermis were reduced in *spo^1^* and *dib^2^* mutant embryos during DC (Fig. 6E-H; quantifications in Fig. S6E-H), indicating promotion of *ush* expression by 20E signaling.

AP-1 binding motifs were found in four EcR binding regions within the *EcR* locus, including two in the EcR-bound area identified in 20E-treated Kc167 cells (Fig. 5C) (ENCODE Project Consortium, 2012; Gauhar et al., 2009). These motifs were also found in binding regions for Jun and/or Kay (Fos), supporting the idea that EcR is guided to binding sites in a complex with AP-1. *EcR* expression comes on strongly in the amnioserosa during germband retraction in wild type but not in *spo^1^* nor *dib^2^* mutant embryos (Fig. 6I-L; quantifications in Fig. S6I,J), suggesting that EcR operates in a positive feedback loop for 20E-mediated gene expression in the amnioserosa. *RhoGAP71E* expression in wild type is typically restricted to the dorsal vessel during DC, but expression was ectopically induced in the dorsal epidermis of *spo^1^* and *dib^2^* mutants (Fig. 6M-P; quantifications in Fig. S6K,L). Interestingly, although an EcR-bound region within the *RhoGAP7E* locus of 20E-induced Kc167 cells was identified, the ENCODE Project Consortium data showed no binding of EcR to this region, but one instance of Usp binding (Fig. 5D) (ENCODE Project Consortium, 2012; Gauhar et al., 2009). Finally, despite *Mes2* being isolated as a putative EcR-binding gene (Gauhar et al., 2009), loss of 20E had no discernible effects on *Mes2* expression in the amnioserosa or the dorsal vessel (Fig. 6Q-T; quantifications in Fig. S6M-P).

## DISCUSSION

With amnioserosa morphogenesis being an important part of DC, it is critical that the timing and degree of amnioserosa contraction is properly modulated and is synchronized with the morphogenesis of the surrounding epidermis. Here, we provide evidence that Dpp secreted from the leading edge epidermis informs the amnioserosa that epidermal morphogenesis is commencing by turning on the expression of *spo* in the extraembryonic tissue. This in turn leads to 20E production, which can then regulate the expression of DC participants in the amnioserosa and nearby tissues such as the dorsal epidermis by promoting complex formation between EcR and the AP-1 transcription factor subunit, Jun, at genomic binding regions that contain AP-1 motifs but no EcREs. The most commonly regulated tissue observed in this study is the amnioserosa, with six of the nine genes examined showing modulation by 20E signaling in the tissue. This is not surprising as the amnioserosa has the highest levels of 20E, EcR, and nuclear EcR-Jun complexes during germband retraction and DC.

We identified three patterns of gene expression in the amnioserosa of wild type embryos, which may result from differing contributions from JNK and 20E signaling. The first pattern, seen with *RhoGAP71E* and *Mes2*, is modest gene expression before the start of germband retraction, which is likely driven by the JNK pathway, that quickly disappears as germband retraction begins, presumably as JNK is shut down in the amnioserosa (Reed et al., 2001). An effect of 20E signaling on this expression pattern is not apparent. The second pattern, seen with *jar* and *zasp52*, is strong expression prior to germband retraction onset that again is likely driven by JNK, but shuts down by mid-germband retraction. This expression pattern requires EcR-mediated repression, as loss of 20E signaling causes aberrant persistence of gene expression in the amnioserosa into DC. The third pattern, seen with *jup*, *zip*, *ush*, and *EcR*, is persistent expression throughout germband retraction and sometimes into DC. This expression pattern requires EcR-mediated activation, as loss of 20E signaling causes premature termination of gene expression in the amnioserosa. In this situation, there may be a ‘hand off’ in regulation where EcR takes control from the AP-1 transcription factor as the JNK pathway is shut down. Notably, EcR does not impart a huge influence on promoting gene expression in the epidermis, which is JNK-dependent, likely because 20E levels are too low. This is supported by treatment of wild type embryos with exogenous 20E, which greatly increases epidermal *zip* expression. We do, however, provide evidence of 20E-mediated repression of gene expression in the epidermis, as seen with *cbt* and *RhoGAP71E*.

Similar to other work, we show that EcR can both positively and negatively regulate gene expression. For example, mammalian estrogen has been shown to activate some genes through AP-1 while repressing others (Björnström and Sjöberg, 2005). Future work will be aimed at determining the composition of EcR-containing complexes at EcR-AP-1 binding regions, and understanding how they can activate or repress gene expression. For example, we have yet to establish roles for the EcR binding partner, Usp, or the Kay (Fos) subunit of the AP-1 transcription factor for these modes of regulation, though ChIP-seq data and our pull-down assays suggest that they may somehow be involved (ENCODE Project Consortium, 2012; Shlyueva et al., 2014). Alternatively, 20E-mediated enhancer repression may be entirely independent of EcR binding, instead involving Ecdysone-induced protein 74 (Eip74) in an unknown mechanism (Shlyueva et al., 2014).

One matter that remains unclear is the exact role of the ecdysone steroid hormone in 20E signaling through EcR-AP1 binding regions. Canonically, 20E activates EcR, which can then form a heterodimer with Usp and bind to EcREs (Dobens et al., 1991; Yao et al., 1993). EcR-W650A, which blocks endogenous EcR from dimerizing with Usp and consequently inhibits expression at EcREs, had no effect on the transcription of the JNK-responsive genes *jar*, *jup*, *zasp52*, and *zip*. In embryos mutant for *spo*, which lack 20E production, PLA complexes between EcR and Jun were abolished in the amnioserosa. However, as EcR itself is also regulated by this non-canonical 20E signaling pathway, we are unable to determine if the absence of PLA complexes in *spo* mutants is due to a loss of 20E production that may promote binding between EcR and Jun, or just solely due to the loss of EcR expression. Pull-down assays between tagged versions of EcR and Jun indicate that direct binding between the two proteins is not enhanced in the presence 20E *in vitro*. But other proteins may be required to modulate this binding. We do, however, observe in wild type embryos that PLA complexes between EcR and Jun are predominately nuclear in the amnioserosa where 20E levels are high, but are mostly cytoplasmic in the epidermis where 20E levels are low. Interestingly, 20E-treatment of embryos causes a shift in the subcellular localization of the EcR-Jun PLA complexes from the cytoplasm to the nucleus in the epidermis. Thus, at the very least, our work indicates that the ecdysone steroid hormone plays a role in the nuclear translocation of EcR-Jun complexes to regulate gene expression.

We have demonstrated that 20E signaling, acting through EcR-AP-1 binding regions, allows for more refined modulation of gene expression than the JNK pathway on its own. This mode of regulation presumably acts elsewhere during development when and where 20E and JNK signaling overlap, which is supported by ChIP-seq data done in different cells, tissues, and developmental stages (ENCODE Project Consortium, 2012; Shlyueva et al., 2014). Alternate tissues to study in the future may be the larval imaginal wing disc, where EcR has been shown to bind to non-canonical ecdysone target genes (Uyehara and McKay, 2019), or the larval salivary gland, where AP-1 is required for 20E-triggered cell death (Lehmann et al., 2002).

Finally, an interesting issue raised by our work is whether it informs us about the origin of steroid hormone-AP-1 interactions. We noticed that EcR-AP-1 binding regions, which apparently recruit EcR and AP-1 transcription factor subunits to DNA, tended to occur in large introns. Indeed, the candidate genes from our screen (listed in Table S1) were on average twice the size of the average *Drosophila* gene (i.e. 22 kb compared to 11 kb). The large introns of the genes containing EcR-AP-1 binding regions may have provided an ideal setting for the emergence of these regulatory sequences by allowing transcription factors to experiment with their DNA binding, which could be followed by the evolution of protein–protein interactions between transcription factors fortuitously finding themselves as neighbors on DNA. This could be a mechanism for convergent evolution of steroid hormone–receptor interactions. We examined the distributions of consensus AP-1 binding motifs and ChIP-seq data for several large genes including *brn-1* (Fig. 5E). Such genes had many ChIP-seq peaks scattered throughout their introns that showed little overlap with AP-1 binding motifs, suggesting that many of the ChIP-seq peaks represent spurious interactions and/or binding to non-consensus sequences (ENCODE Project Consortium, 2012; Spivakov, 2014). In the absence of molecular comparisons between *Drosophila* and vertebrate steroid hormone receptor-AP-1 complexes, it remains uncertain if our results support an ancient origin of interactions between these transcription factor families.

## MATERIALS AND METHODS

### Fly stocks

Flies were maintained at 25°C under standard conditions (Ashburner and Roote, 2007). *w^1118^* was used as a wild type control strain unless otherwise stated. *spo^Z339^* was a kind gift from M. O’Connor, Department of Genetics, Cell Biology and Development, University of Minnesota, MN, USA (Ono et al., 2006), and *ubi-DE-cadherin-GFP* was generously provided by H. Oda, Laboratory of Evolutionary Cell and Developmental Biology, Biohistory Research Hall, Japan (Oda and Tsukita, 2001). All other stocks were obtained from the Bloomington *Drosophila* Stock Center, IN, USA.

### Live imaging of embryos

Embryos were prepared for live imaging using the hanging drop protocol (Reed et al., 2009), and imaged with a Nikon Eclipse 90i microscope with a Nikon D-Eclipse C1 scan head. Images were saved as animated projections using Nikon EZ-C1 software and further processed with ImageJ (NIH). A *ubi-DE-cadherin-GFP* transgene was expressed in all embryos to visualize morphology (Oda and Tsukita, 2001).

### 20E treatment of embryos

Embryonic treatment with exogenous 20E was performed as previously described (Kozlova and Thummel, 2003). Embryos were collected for 6 h (Rothwell and Sullivan, 2007a), then cultured for another 4 h in MBIM, supplemented with 5×10^−6^ M 20E (H5142, Sigma-Aldrich) dissolved in ethanol, prior to fixation (Rothwell and Sullivan, 2007b). Control embryos, done in parallel, were subjected to the same treatment but replacing 20E in ethanol with ethanol alone.

### FISH

Detection of transcripts *in situ* by FISH was performed as described previously (Lécuyer et al., 2008). cDNA templates used to make full-length antisense probes were obtained from the *Drosophila* Genomics Resource Center. Fluorescently stained embryos were imaged on a Nikon A1R laser scanning confocal microscope with NIS-Elements software, and the images were processed with Adobe Photoshop. Mutant stocks were re-balanced over GFP-tagged balancers allowing for homozygotes to be selected based on the absence of GFP signal. Heterozygous siblings, which were treated under identical conditions within the same tube, served as controls. For transgenic analysis, homozygous *UAS*-transgene-bearing males were crossed to homozygous *Gal4*-bearing virgin females ensuring that all progeny carried one copy of each. In cases where either the *Gal4* or *UAS*-transgenic stock was homozygous lethal, the stock was also re-balanced over a GFP-containing balancer. In subsequent crosses, GFP-negative embryos carried both the *Gal4* and *UAS*-transgene, whereas GFP-positive embryos lacked either the *Gal4* or *UAS*-transgene and, therefore, had no transgenic expression.

### Quantification of FISH signal

#### FISH signal in the amnioserosa

Expression levels in the amnioserosa were quantified by counting the number of pixels that made up the fluorescent signals derived from FISH. Heterozygous siblings of the homozygous mutant embryos served as controls for each stain, as they were treated under identical conditions within the same tube. For each embryo, the z-stacked confocal image was first converted to grayscale with Adobe Photoshop. The amnioserosa was next hand-selected with the Lasso tool, and the surface area of the tissue was measured as pixel surface area. The selection was next copied and pasted into a new file, then opened under ImageJ (NIH). The selection was inverted and the threshold was adjusted to create a black and white image, where black represented the FISH signal and white represented the background. The FISH signal was then measured as the total number of black pixels. To standardize the measurement between embryos, the number of black pixels was divided by the pixel surface area of the amnioserosa. Data were expressed as absolute values, and presented as means±s.e.m.. Student's *t*-tests were performed for all statistical comparisons using GraphPad. Note that the parameters used for quantification were kept constant within data sets. See Fig. S7A for examples of the quantification.

#### FISH signal in the DME cells

Expression levels in the DME cells were quantified by measuring the intensities of the fluorescent signals derived from FISH. Heterozygous siblings of the homozygous mutant embryos served as controls for each stain, as they were treated under identical conditions within the same tube. For each embryo, the z-stacked confocal image was first converted to grayscale with Adobe Photoshop. A section of leading edge epidermis corresponding to one embryonic segment was next selected using the Rectangular Marquee tool with a fixed selection size. The fluorescence intensity of the FISH signal was then measured as mean gray value. Multiple sections of leading-edge epidermis were analyzed per embryo. Data were expressed as absolute values, and presented as means±s.e.m.. Student's *t*-tests were performed for all statistical comparisons using GraphPad. Note that the parameters used for quantification were kept constant within data sets. See Fig. S7B for an example of the quantification.

### PLA

PLA was performed as previously described but with modifications (Thymiakou and Episkopou, 2011). Fixed embryos (Rothwell and Sullivan, 2007a,b) were blocked for 1 h with 1% BSA (in PBT: 3 mM NaH_2_PO_4_·H_2_O, 7 mM Na_2_PO_4_, 1.3 M NaCl, 0.1% Triton X-100, pH 7.0). Next, the embryos were incubated with 1:5 mouse anti-EcR (DDA2.7, Developmental Studies Hybridoma Bank) (Talbot et al., 1993) and 1:25 rabbit anti-Jun (sc-25763, Santa Cruz Biotechnology) primary antibodies in 1% BSA overnight at 4°C. For negative controls, either mouse anti-EcR was omitted or rabbit anti-Jun was replaced with rabbit anti-pMad (Persson et al., 1998). After three PBT washes for 10 min each, the embryos were incubated with 1:5 dilutions of anti-rabbit PLUS (DUO92002, Sigma-Aldrich) and anti-mouse MINUS (DUO92004, Sigma-Aldrich) PLA probes in 1% BSA for 2 h at 37°C. The embryos were subsequently washed twice with Wash A for 5 min each, then incubated in Ligation reagent (DUO92008, Sigma-Aldrich) for 1 h at 37°C. Following two washes with Wash A for 2 min each, the embryos were incubated in Amplification reagent (DUO92008, Sigma-Aldrich) for 2 h at 37°C. After two Wash A washes for 2 min each, the embryos were incubated with 1:200 FITC-conjugated anti-mouse or anti-rabbit secondary antibody (Jackson ImmunoResearch) in 1% BSA for 1 h. Finally, the embryos were washed twice with Wash B for 10 min each, followed by a single wash with 0.01x Wash B for 1 min, then stored in Duolink In Situ Mounting Medium with DAPI (DUO82040, Sigma-Aldrich) at −20°C until ready for confocal imaging.

### GST pull-down assays

Preparation of tagged proteins was performed as described previously (Rebay and Fehon, 2009). The following cDNA clones, obtained from the *Drosophila* Genomics Resource Center, IN, USA were used: *EcR* (RE06878), *jun* (LD25202), *usp* (LD09973), and *kay* (LP01201). Full-length coding regions were amplified and inserted in frame into pET-28a(+) (69864-3, MilliporeSigma) and/or pGEX-4T-1 (28-9545-49, GE Healthcare) to create N-terminal, His- and GST-tagged constructs, respectively. The constructs were transformed into BL21(DE3) competent cells (C2527, New England Biolabs) for expression.

Pull-downs were standardized by adding an equivalent amount of bait protein (i.e. the GST-tagged protein from the bacterial soluble protein fraction) to an equal volume of prey protein (i.e. the His-tagged protein from the bacterial soluble protein fraction). The volume was then topped up to 500 µl with Buffer A (20 mM Tris, 1 mM MgCl_2_, 150 mM NaCl, 0.1% NP-40, 10% Glycerol, 1x cOmplete protease inhibitor cocktail, pH 8.0), and the mix was incubated for 1.5 h at 4°C. In the meantime, 25 µl of Glutathione Sepharose 4B (17-0756-01, GE Healthcare) was blocked with 1% BSA (in Buffer A) for 1 h at 4°C. The mix was then added to the blocked beads and incubated for another 1.5 h at 4°C. For testing 20E-mediated effects on binding, the appropriate amount of 20E (H5142, Sigma-Aldrich) was also included. Following three washes with Buffer A, bound proteins were denatured and fractionated by SDS-PAGE. The presence of His-tagged, prey proteins was determined by immunoblotting with the use of the following primary antibodies: 1:150 mouse anti-EcR (DDA2.7, DSHB) (Talbot et al., 1993) and 1:1000 rabbit anti-Jun (sc-25763, SCBT). Both antibodies were diluted in 1% milk (in TBST: 1.5 M Tris, 0.5 M NaCl, 0.1% Tween 20, pH 7.5). Peroxidase-conjugated secondary antibodies (Vector Laboratories) were used at a 1:2000 dilution in 1% milk, and signal was detected with BM Chemiluminescence Blotting Substrate (11500694001, Roche).

## Supplementary Material

Supplementary information
