# Supplementary material for: 20-hydroxyecdysone (20E) signaling regulates amnioserosa morphogenesis during Drosophila dorsal closure: EcR modulates gene expression in a complex with the AP-1 subunit, Jun
Source: Biol Open. 2021 Aug 17;10(8):bio058605. doi: 10.1242/bio.058605 (PMC8411571; doi:10.1242/bio.058605)
Supplement: Supplementary information [file biolopen-10-058605-s1.pdf]

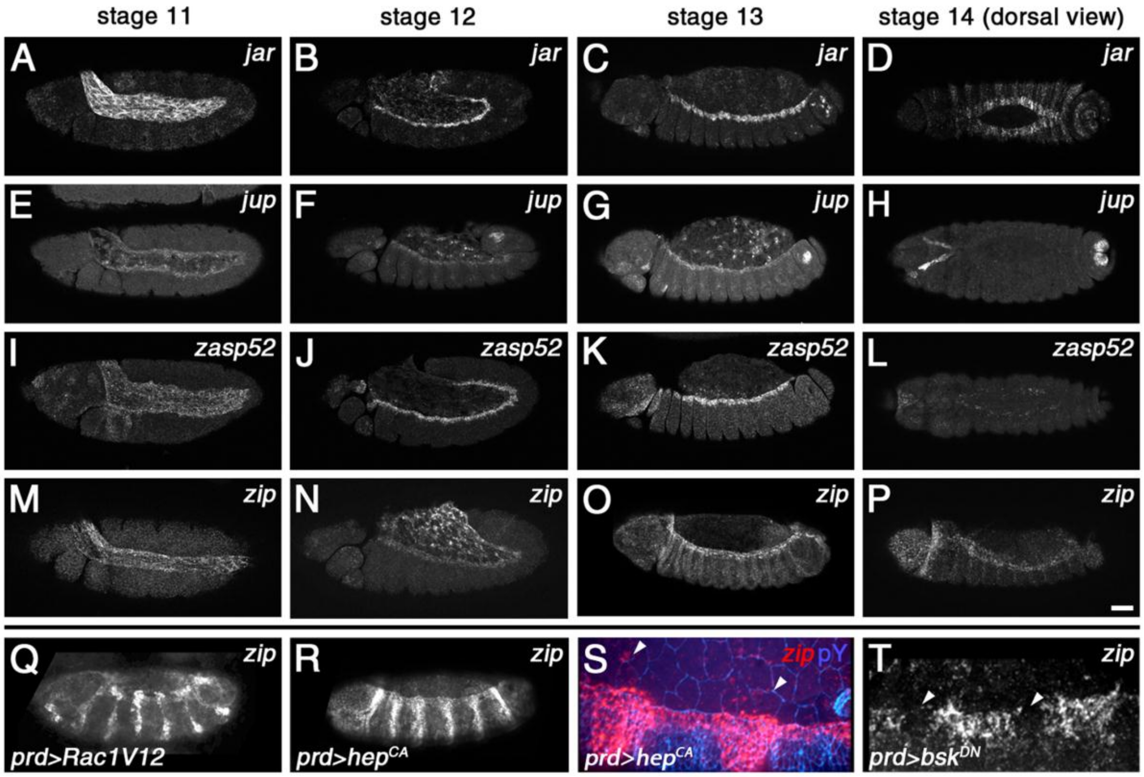

Fig. S1. FISH showing endogenous expression of JNK-responsive genes during late embryonic development. (A-P) Shown are progressively older wild-type embryos from left to right depicting early germband retraction (stage 11), mid-germband retraction (stage 12), mid-DC (stage 13), and late DC (stage 14). *jar*, *jup*, *zasp52* and *zip* are all expressed in the amnioserosa prior to germband retraction (A,E,I,M). However, as *jup* and *zip* expression is still present in the amnioserosa during germband retraction (F,N), *jar* and *zasp52* expression promptly shuts off (B,J). *jup* expression persists in the amnioserosa until late DC (G,H), whereas *zip* expression is almost absent as DC begins (O). All four genes show expression in the dorsal-most epidermal (DME) cells (C,G,K,O), which flank the amnioserosa, but fades as DC progresses (D,H,L,P). (Q-T) FISH

Biology Open • Supplementary information

experiments demonstrating that the expression of *zip* is regulated by the JNK pathway. Activation of the JNK pathway through expression of transgenic Rac1V12 (Q) or Hep^{CA} (R,S) in *paired* (*prd*) stripes elevates *zip* expression in the epidermis (Q,R). Ectopic *zip* expression in the amnioserosa (arrowheads) can also be observed (S). Panel shows high magnification view of a merge between *zip* FISH (red) and anti-phosphotyrosine (pY) staining (blue), which marks cell membranes. Inhibition of the JNK pathway through expression of transgenic Bsk^{DN} in *prd* stripes causes loss of *zip* expression (T). Panel shows high magnification view of gaps in *zip* expression in the epidermis (arrowheads). Scale bar represents 50µm (P).

Biology Open • Supplementary information

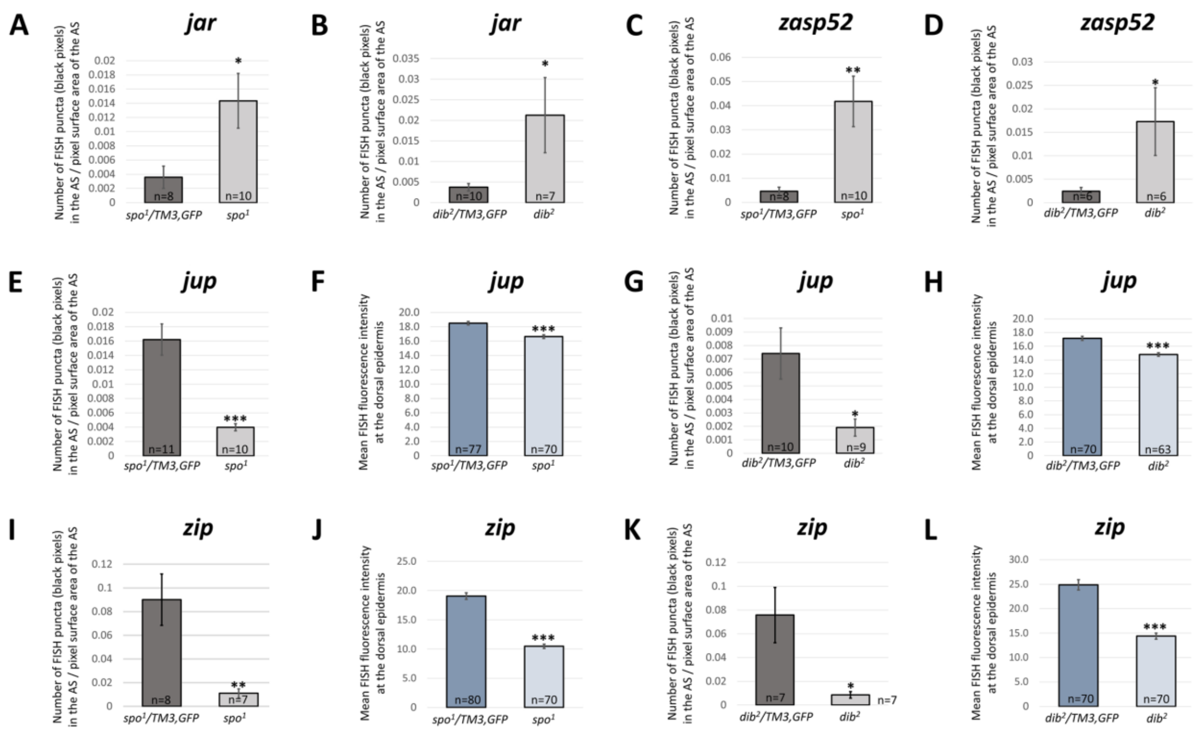

Fig. S2. Quantification of the effects of *spo* and *dib* mutations on the expression of JNK-responsive genes. Representative FISH stains are shown in Fig. 2. Stages of embryos analyzed ranged from late germband retraction (stage 12) to mid-DC (stage 13). For amnioserosa (AS) measurements, the total number of embryos (n) analyzed for each genotype is displayed in the bar graphs. For epidermis measurements, the total number of dorsal epidermal segments (n) analyzed for each genotype is also displayed. (A,B) Quantification of *jar* FISH signals in the amnioserosa. (C,D) Quantification of *zasp52* FISH signals in the amnioserosa. (E-H) Quantification of *jup* FISH signals in the amnioserosa (E,G) and dorsal epidermis (F,H). For epidermis measurements, seven segments were analyzed per embryo. (I-L) Quantification of *zip* FISH signals in the amnioserosa (I,K) and dorsal epidermis (J,L). For epidermis measurements, ten segments were analyzed per embryo. * $p < 0.05$, ** $p < 0.01$, *** $p < 0.001$.

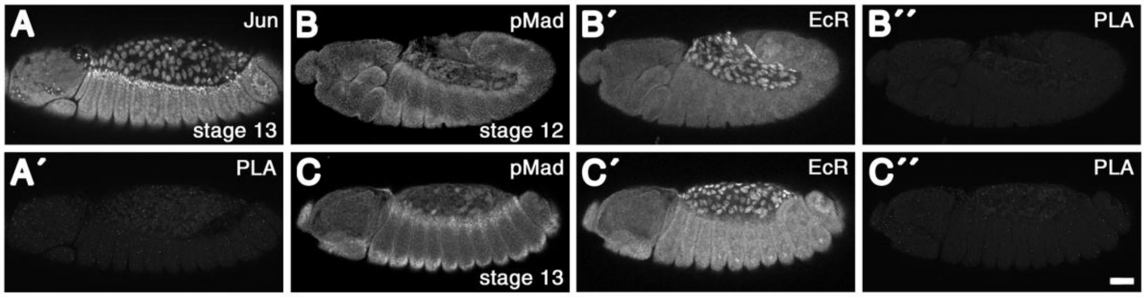

Fig. S3. Negative control experiments for PLA. (A,A') PLA experiment in which anti-EcR antibody was omitted. While there is robust anti-Jun staining (A), there are no clusters of PLA signals in the amnioserosa (A'). (B-C'') PLA experiment in which anti-Jun antibody was replaced with anti-pMad. The anti-pMad antibody detects another transcription factor that drives gene expression in the amnioserosa and dorsal epidermis. Despite strong anti-pMad (B,C) and anti-EcR (B',C') staining during germband retraction (B-B'') and DC (C-C''), there are no observable PLA signals (B'',C''). Scale bar represents 50μm (C'').

Biology Open • Supplementary information

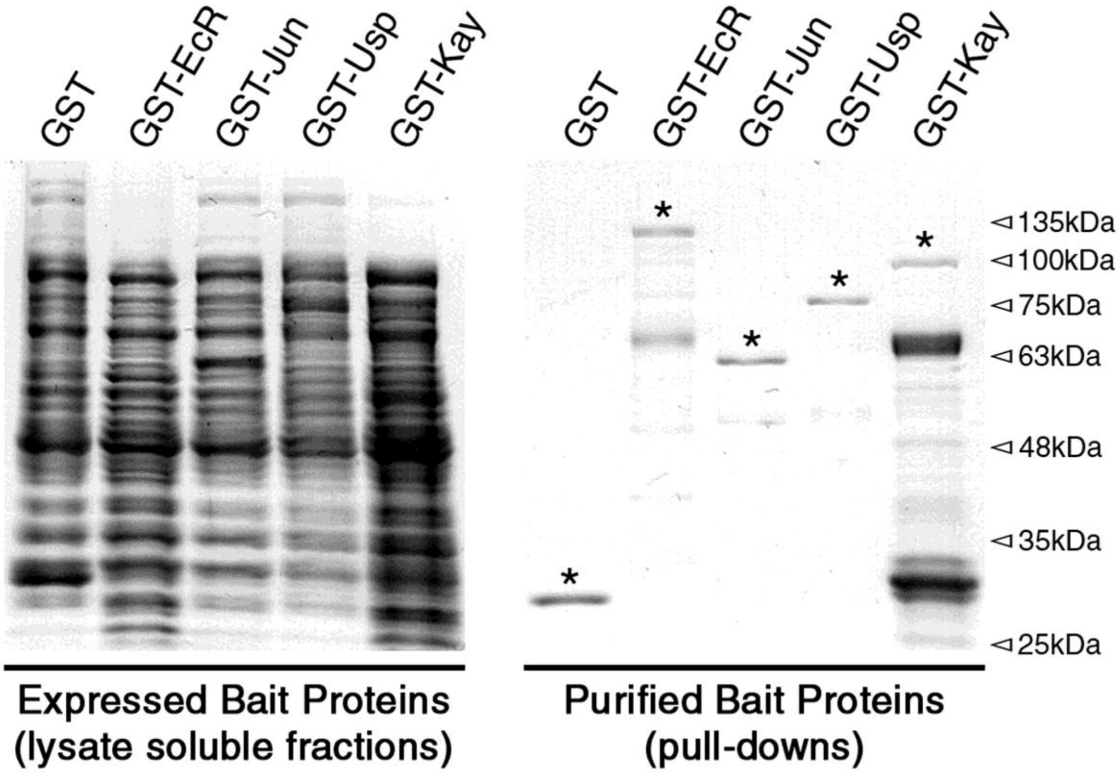

Fig. S4. Western blot analysis of the levels of each bait protein used in the pull-down assays. Shown are gels stained with Coomassie Brilliant Blue. GST fusion (bait) proteins were expressed in BL21(DE3) bacterial cells (left, lysate soluble fraction) and purified with Glutathione Sepharose (right). Input percentages are as follows: GST (100%), GST-EcR (15%), GST-Jun (75%), GST-Usp (75%), and GST-Kay (4.3%). Asterisks denote bands of interest for GST (expected size = 27.9kDa), GST-EcR (120.2kDa), GST-Jun (57.7kDa), GST-Usp (81.9kDa), and GST-Kay (89.5kDa). Same amounts were used in the experimental pull-downs shown in Fig. 4H-K.

Biology Open • Supplementary information

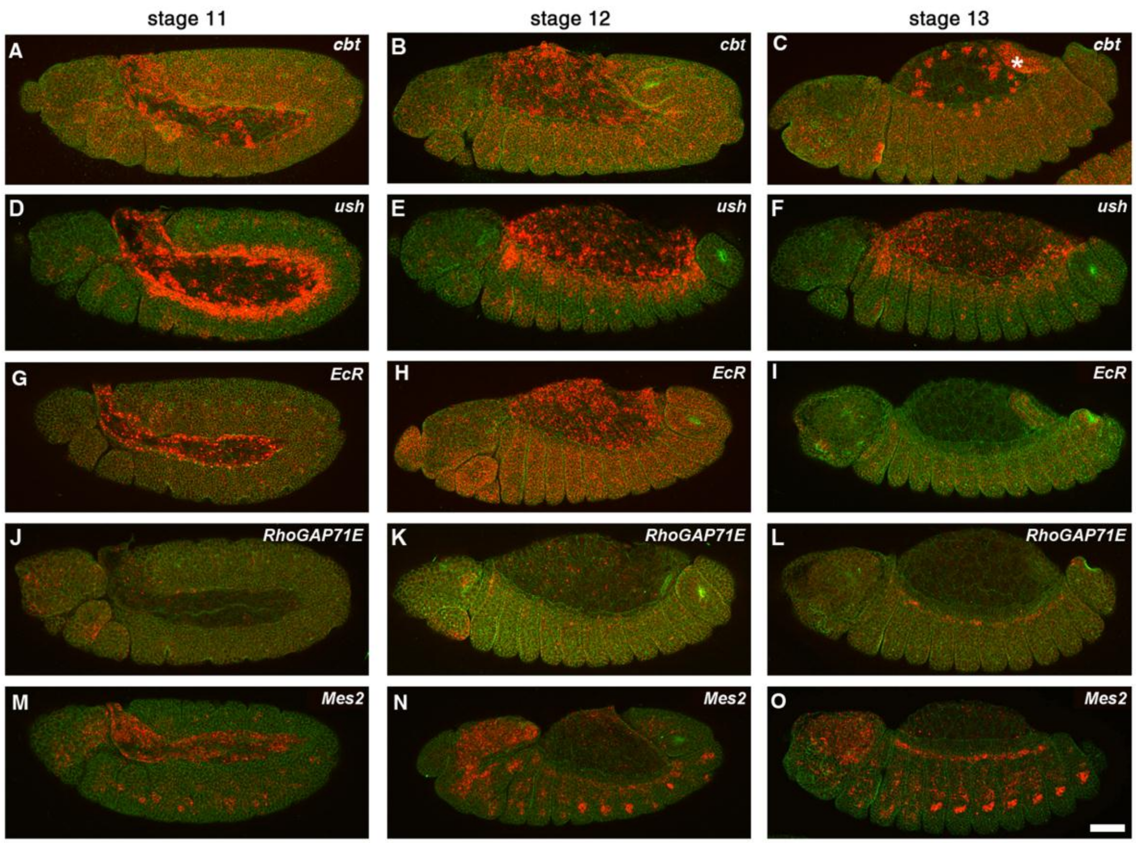

Fig. S5. FISH showing endogenous expression of genes bearing putative EcR-AP-1 binding regions. Shown are merged images of FISH signal (red) and anti-phosphotyrosine (pY) staining (green), which marks cell membranes to help distinguish the boundary between the amnioserosa and epidermis. Wild-type embryos are progressively older from left to right, and depict early germband retraction (stage 11), mid-germband retraction (stage 12), and mid-DC (stage 13). (A-C) *cbt* is expressed strongly in the amnioserosa, yolk sac nuclei, and hindgut (asterisk), with lower levels present in the epidermis, during germband retraction (A,B) and DC (C). (D-F) *ush* is expressed predominately in peripheral amnioserosa cells and the dorsal epidermis during germband retraction (D,E)

Biology Open • Supplementary information

and DC (F). (G-I) *EcR* expression is present in the amnioserosa during germband retraction (G,H), but is promptly turned off by the onset of DC (I). (J-L) *RhoGAP71E* expression is shut down in the amnioserosa during germband retraction (J,K), but appears in the dorsal vessel by the beginning of DC (L). (M-O) *Mes2* has a similar expression pattern as *RhoGAP71E*, but is also expressed in head tissues and ventrally in oenocytes. Scale bar represents 50µm (O).

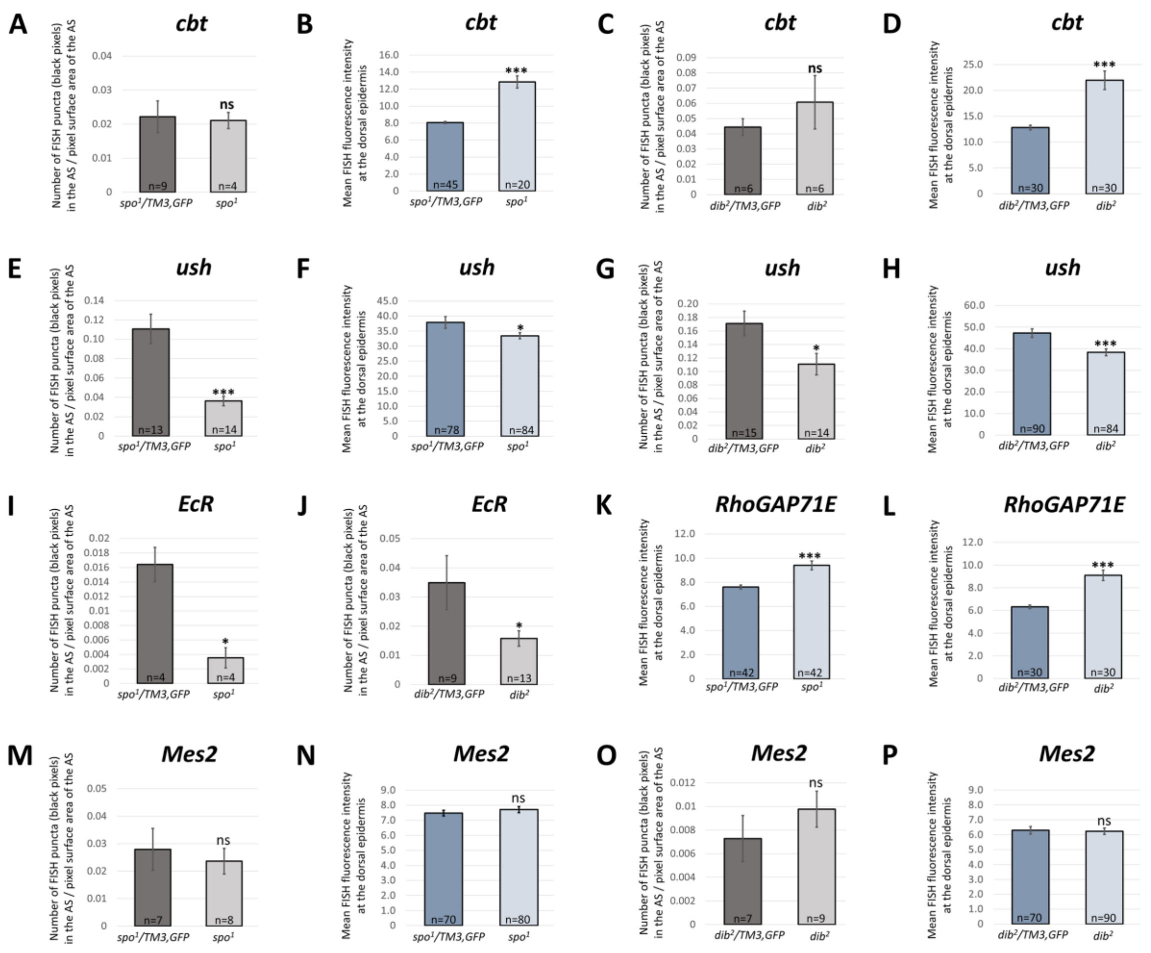

Fig. S6. Quantification of the effects of *spo* and *dib* mutations on the expression of genes bearing putative EcR-AP-1 binding regions.

Representative FISH stains are shown in Fig. 6. Stages of embryos analyzed ranged from late germband retraction (stage 12) to mid-DC (stage 13). For amnioserosa (AS) measurements, the total number of embryos analyzed for each genotype (n) is displayed in the bar graphs. For epidermis measurements, the total number of dorsal epidermal segments analyzed for each genotype (n) is also displayed. (A-D) Quantification of *cbt* FISH in the amnioserosa (A,C) and

dorsal epidermis (B,D). For epidermis measurements, five segments were analyzed per embryo. (E-H) Quantification of *ush* FISH in the amnioserosa (E,G) and dorsal epidermis (F,H). For epidermis measurements, six segments were analyzed per embryo. (I,J) Quantification of *EcR* FISH in the amnioserosa. (K,L) Quantification of *RhoGAP71E* FISH in the dorsal epidermis. Six segments were analyzed per embryo. (M-P) Quantification of *Mes2* FISH in the amnioserosa (M,O) and dorsal epidermis (N,P). For epidermis measurements, ten segments were analyzed per embryo. * $p < 0.05$, ** $p < 0.01$, *** $p < 0.001$.

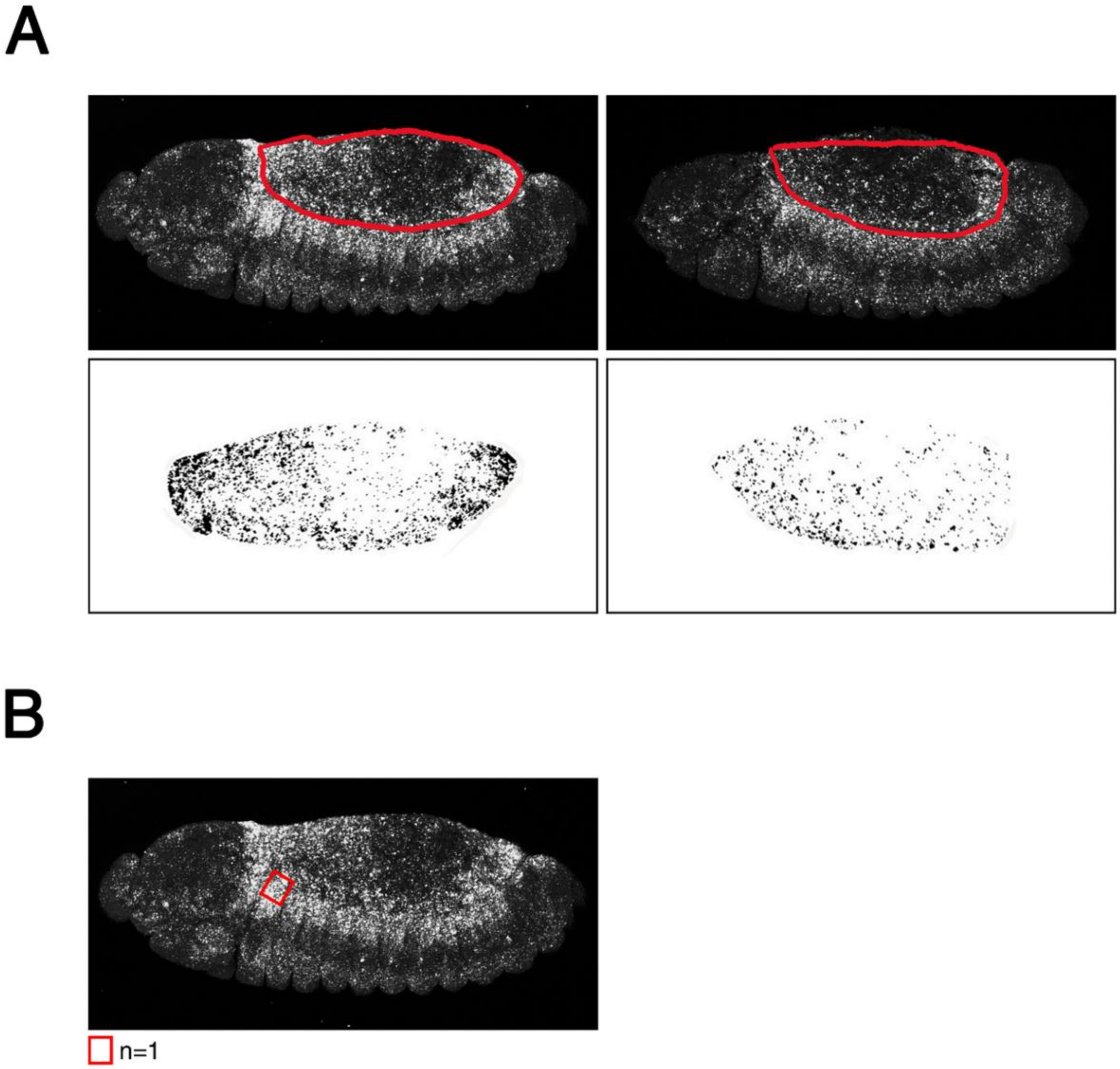

Fig. S7. Examples of FISH signal quantification. (A) Transcription levels in the amnioserosa were quantified by counting the number of pixels that made up the fluorescent signals derived from FISH. For each embryo, the z-stacked confocal image was first converted to grayscale with Adobe Photoshop (top panels). The amnioserosa was next hand-selected with the Lasso tool (selection boundaries symbolized in red), and the surface area of the tissue was measured as pixel

Biology Open • Supplementary information

surface area. The selection was next copied and pasted into a new file, then opened under ImageJ (NIH). The selection was inverted and the threshold was adjusted to create a black and white image, where black represented the FISH signal and white represented the background (bottom panels). The FISH signal was then measured as the total number of black pixels. To standardize the measurement between embryos, the number of black pixels was divided by the pixel surface area of the amnioserosa. (B) Transcription levels in the DME cells were quantified by measuring the intensities of the fluorescent signals derived from FISH. For each embryo, the z-stacked confocal image was first converted to grayscale with Adobe Photoshop. A section of leading edge epidermis corresponding to one embryonic segment was next selected using the Rectangular Marquee tool with a fixed selection size (selection boundary symbolized in red). The fluorescence intensity of the FISH signal was then measured as mean gray value. Multiple sections of leading edge epidermis were analyzed per embryo. See Materials and Methods for more details.

Table S1.: Genes near or containing putative EcR-AP-1 binding regions consisting of at least four AP-1 binding motifs (TGANTCA) but no EcREs.

Chromosome	Gene Symbol	Annotation Symbol	DC Gene?	Expressed in Amnioserosa?	Expressed in Dorsal Tissue?	References
X	*CG12535* and/or *CG14269*	CG12535 and/or CG14269	?	?	?	-
X	*Agpat1* and/or *CG32647*	CG3812 and/or CG32647	?	?	?	-
X	*IP3K2*	CG45017	?	N	**Y**	BDGP
2L	*cbt*	CG4427	Y	Y	**Y**	Muñoz-Descalzo et al., 2005; Belacortu et al., 2011; BDGP
2L	*ush*	CG2762	Y	Y	Y	Lada et al., 2012; BDGP
2L	*Kr-h1*	CG45074	?	N	Y	BDGP
2L	*Akap200*	CG13388	?	Y	Y	BDGP
2L	*Pect*	CG5547	?	Y	Y	BDGP
2L	*B4*	CG9239	?	?	?	-
2L	*CG5953*	CG5953	?	Y	N	BDGP
2L	*MESR3*	CG15162	?	?	Y	BDGP
2L	*brat*	CG10719	?	N	N	BDGP
2L	*CdGAPr*	CG10538	?	N	?	Sagnier et al., 2000
2L	*sky*	CG9339	?	?	?	-
2L	*step*	CG11628	Y	Y	Y	West et al., 2017; BDGP
2R	*EcR*	CG1765	Y (GBR)	Y	Y	Kozlova and Thummel, 2003
2R	*chk*	CG3409	?	Y	Y	BDGP
2R	*Roc2*	CG8998	?	?	?	-
2R	*CG17574*	CG17574	?	?	?	-
2R	*shot*	CG18076	Y	N	Y	Strumpf and Volk, 1998; Takacs et al., 2017
2R	*Cp1*	CG6692	?	Y	Y	BDGP
2R	*Rho1*	CG8416	Y	Y	Y	Harden et al., 1999; BDGP
2R	*GstE* gene cluster		?	?	?	-
2R	*MFS14*	CG15095	?	Y	Y	BDGP
2R	*ena*	CG15112	Y	Y	Y	Grevengoed et al., 2001; Gates et al., 2007
2R	*CG13868*	CG13868	?	Y	Y	BDGP
2R	*βTub60D*	CG3401	?	N	Y	BDGP
2R	*Mmp1*	CG4859	?	N	Y	Page-McCaw et al., 2003; BDGP
2R	*zip*	CG15792	Y	Y	Y	Young et al., 1993; Zahedi et al., 2008; BDGP
3L	*promL*	CG7740	?	Y	Y	-
3L	*Ack* and/or *Chd64*	CG14992 and/or CG14996	Y	Y	Y	Sem et al., 2002

Biology Open • Supplementary information

3L	*h*	CG6494	?	N	Y	BDGP
3L	*CG6685*	CG6685	?	?	?	-
3L	*CG32091*	CG32091	?	?	?	-
3L	*Frl*	CG32138	?	?	?	-
3L	*RhoGAP71E*	CG32149	?	N	N	BDGP
3L	*CG5151*	CG5151	?	?	?	-
3L	*CG5290*	CG5290	?	N	N	BDGP
3L	*Eip75B*	CG8127	?	N	Y	Chavoshi et al., 2010
3L	*Rcd2*	CG4786	?	?	?	-
3L	*Mes2*	CG11100	Y	Y	Y	Zimmermann et al., 2006; BDGP
3R	*kra*	CG2922	?	Y	Y	BDGP
3R	*CG8312*	CG8312	?	Y	Y	BDGP
3R	*fabp* and/or *Mrp4*	CG6783 and/or CG14709	?	?	?	-
3R	*GstD* gene cluster		?	?	?	-
3R	*red*	CG12207	?	N	N	BDGP
3R	*Xrp1*	CG17836	?	Y	Y	BDGP
3R	*SNF4Aγ*	CG17299	?	Y	Y	BDGP
3R	*InR*	CG18402	Y	N	Y	Fernandez et al., 1995
3R	*Gdh*	CG5320	?	Y	Y	BDGP
3R	*Gprk2*	CG17998	?	?	?	-

DC Gene?: Y – previously shown to be involved in some aspect of DC or germband retraction (GBR); ? – DC role has yet to be determined to our knowledge. Expressed in Amnioserosa/Dorsal Tissue?: Y – previously shown to be expressed in the amnioserosa and/or dorsal tissues such as the dorsal epidermis, yolk sac, and hindgut; N – no expression observed based primarily on *in situ* hybridisation (ISH) results generated by the Berkeley *Drosophila* Genome Project (BDGP) (https://insitu.fruitfly.org/cgi-bin/ex/insitu.pl) (Hammonds et al., 2013; Tomancak et al., 2002; Tomancak et al., 2007); ? – expression has yet to be determined to our knowledge.

Biology Open • Supplementary information

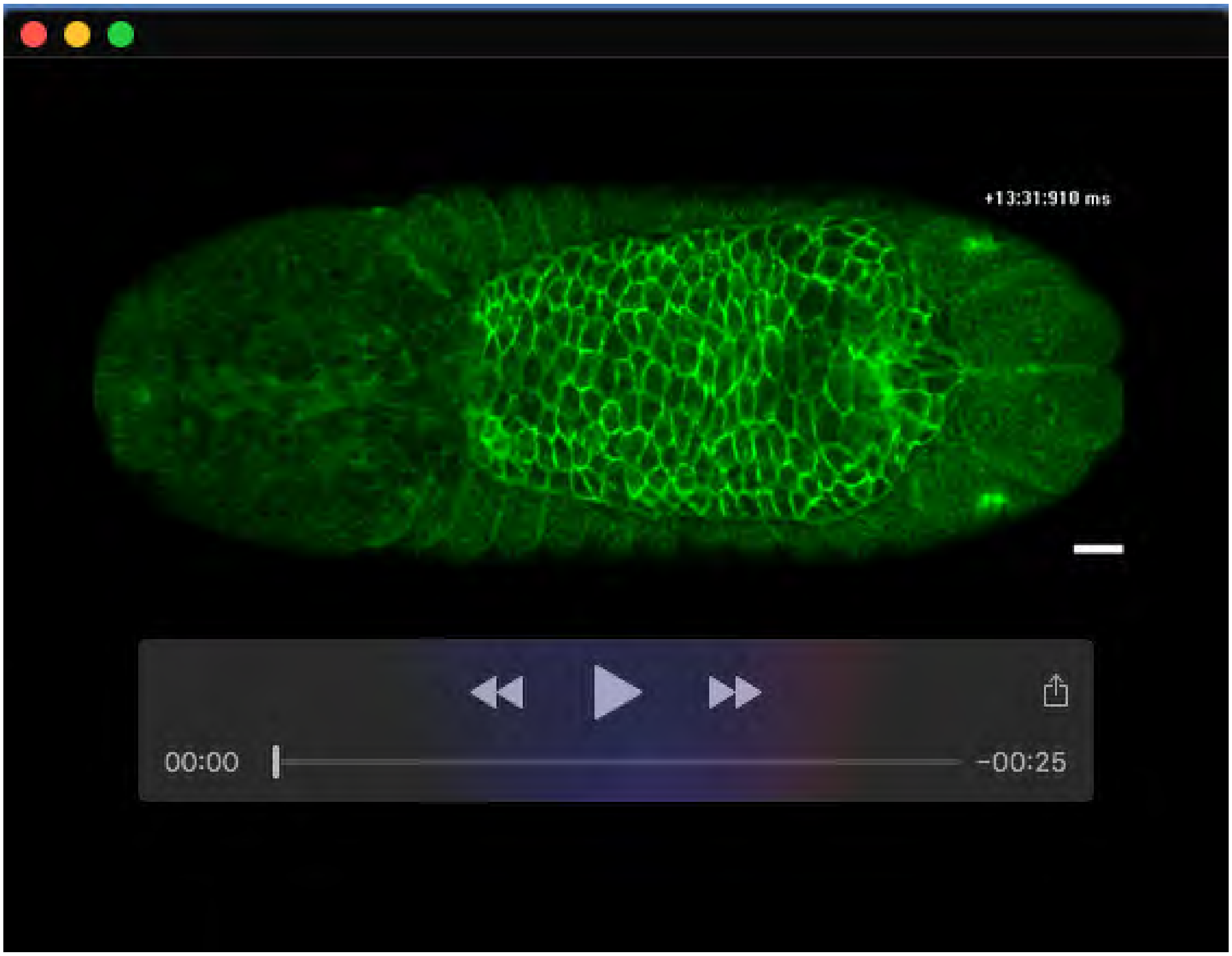

Movie 1. The process of DC shown in a time-lapse movie of a wild-type embryo expressing DE-cadherin-GFP. Each frame is a Z-stack projection. Elapsed time (h:min:s:ms) is shown in the top right. Scale bar represents 20μm. Selected frames from this movie are shown in Fig. 1C,D.

Biology Open • Supplementary information

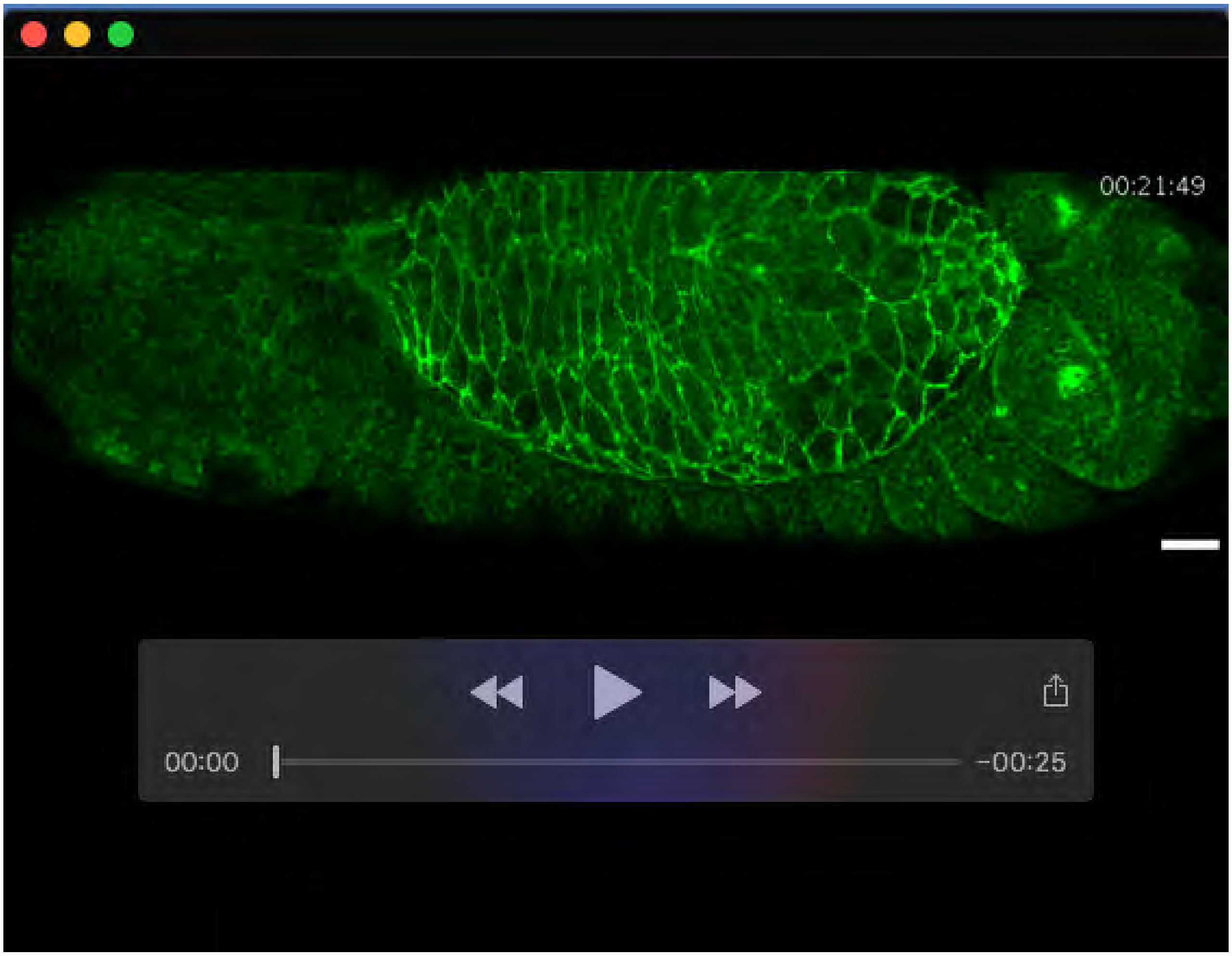

Movie 2. Delay and failure to complete DC shown in a time-lapse movie of a *spo*^{Z339} mutant embryo expressing DE-cadherin-GFP. The body movement indicates completion of somatic musculature innervation, a process that normally occurs following the completion of DC. Each frame is a Z-stack projection. Elapsed time (h:min:s) is shown in the top right. Scale bar represents 20µm. Selected frames from this movie are shown in Fig. 1E,F.

Biology Open • Supplementary information

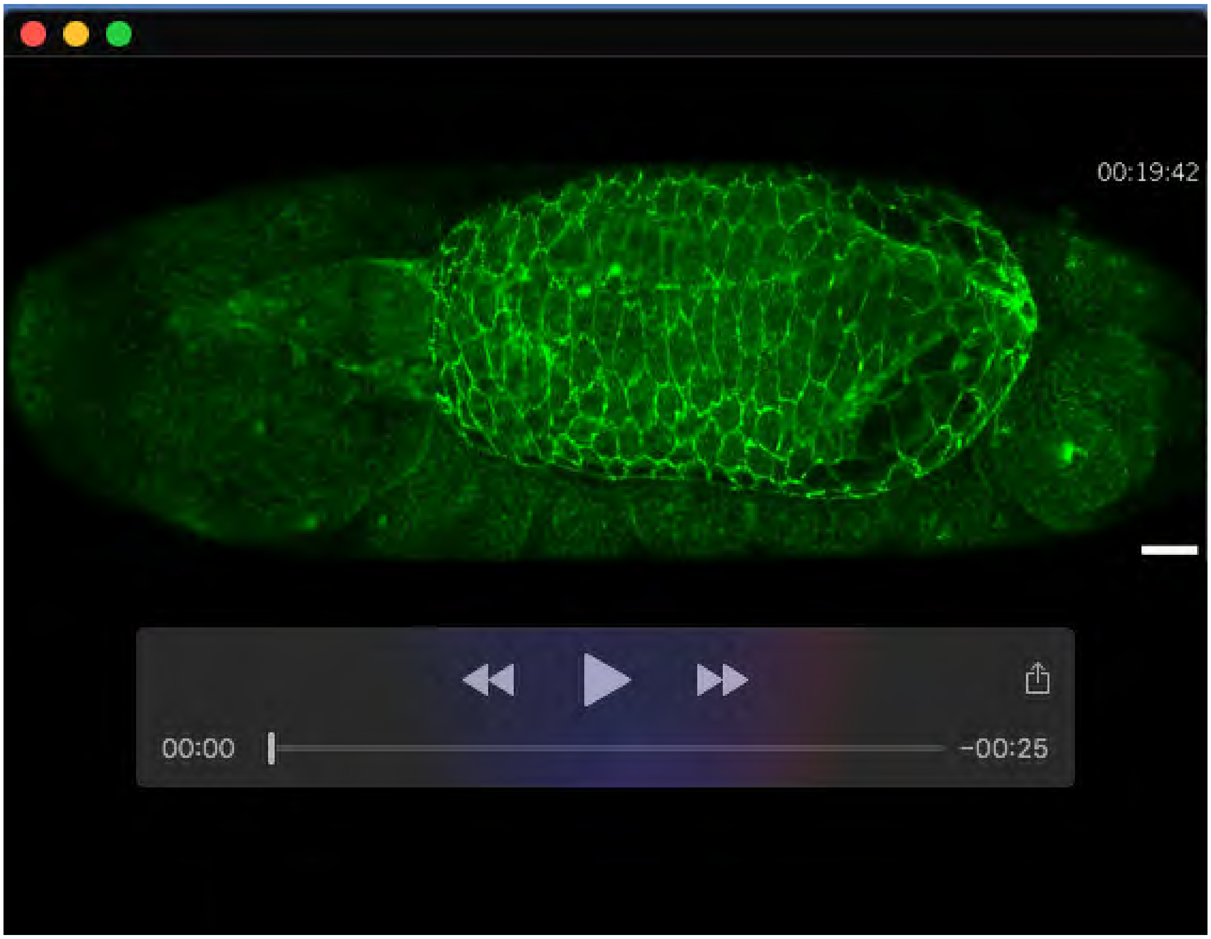

Movie 3. Delay and failure to complete DC shown in a time-lapse movie of a *dib*^{2} mutant embryo expressing DE-cadherin-GFP. The *dib*^{2} phenotype is indistinguishable from that described for *spo*^{Z339} (see Movie S2). Each frame is a Z-stack projection. Elapsed time (h:min:s) is shown in the top right. Scale bar represents 20µm. Selected frames from this movie are shown in Fig. 1G,H.
